# Childhood Learning Disabilities and Atypical Dementia: A Retrospective Chart Review

**DOI:** 10.1371/journal.pone.0129919

**Published:** 2015-06-24

**Authors:** Alon Seifan, Stephanie Assuras, Edward D. Huey, Jesse Mez, Angeliki Tsapanou, Elise Caccappolo

**Affiliations:** 1 Department of Neurology Weill Cornell Medical College, New York, New York, United States of America; 2 Department of Neurology Columbia University, New York, New York, United States of America; 3 Gertrude H. Sergievsky Center, Columbia University, New York, New York, United States of America; 4 Cognitive neuroscience division, Taub Institute for Research on Alzheimer's Disease and the Aging Brain, Columbia University, New York, New York, United States of America; 5 Department of Neurology, Boston University School of Medicine, Boston, Massachusetts, United States of America; 6 Department of Neuropsychology, Columbia University, New York, New York, United States of America; University of California, San Francisco, UNITED STATES

## Abstract

**Objective:**

To further our understanding of the association between self-reported childhood learning disabilities (LDs) and atypical dementia phenotypes (Atypical Dementia), including logopenic primary progressive aphasia (L-PPA), Posterior Cortical Atrophy (PCA), and Dysexecutive-type Alzheimer’s Disease (AD).

**Methods:**

This retrospective case series analysis of 678 comprehensive neuropsychological assessments compared rates of self-reported LD between dementia patients diagnosed with Typical AD and those diagnosed with Atypical Dementia. 105 cases with neuroimaging or CSF data available and at least one neurology follow-up were identified as having been diagnosed by the neuropsychologist with any form of neurodegenerative dementia. These cases were subject to a consensus diagnostic process among three dementia experts using validated clinical criteria for AD and PPA. LD was considered Probable if two or more statements consistent with prior LD were documented within the Social & Developmental History of the initial neuropsychological evaluation.

**Results:**

85 subjects (Typical AD n=68, Atypical AD n=17) were included in the final analysis. In logistic regression models adjusted for age, gender, handedness, education and symptom duration, patients with Probable LD, compared to patients without Probable LD, were significantly more likely to be diagnosed with Atypical Dementia vs. Typical AD (OR 13.1, 95% CI 1.3-128.4). All three of the L-PPA cases reporting a childhood LD endorsed childhood difficulty with language. By contrast, both PCA cases reporting Probable childhood LD endorsed difficulty with attention and/or math.

**Conclusions:**

In people who develop dementia, childhood LD may predispose to atypical phenotypes. Future studies are required to confirm whether atypical neurodevelopment predisposes to regional-specific neuropathology in AD and other dementias.

## Introduction

Adults with a childhood history of specific learning disabilities (LDs) may be more likely to develop atypical phenotypes of dementia, including atypical Alzheimer’s Disease (AD) [1–3]. Typical AD begins with memory symptoms at onset which correlate well with tau pathology in the hippocampus [4]. Atypical AD phenotypes involve focal, primarily non-amnestic presentations, with symptoms that correlate with non-hippocampal tau pathology [5]. For example, when logopenic Primary Progressive Aphasia (L-PPA) is caused by AD pathology, it begins with word-finding difficulty at onset, with tau-related neurodegeneration prominent in language associational cortex [6]. Posterior Cortical Atrophy (PCA) and “Dysexecutive” variants of AD involve visual and dysexecutive symptoms at onset, with prominent neurodegeneration found in visual and frontal association cortices, respectively [7–11]. Non-AD dementia syndromes caused by frontotemporal lobar dementia (FTLD) pathology, including Semantic Dementia (SD), behavioral variant FTD (bvFTD) and non-fluent/agrammatic Primary Progressive Aphasia (aPPA), also exhibit significant phenotypic heterogeneity [12].

The reasons why different brain networks are affected by dementia pathology in different people are unclear. This is a critical research gap because a better understanding of the factors that distinguishes atypical from typical phenotypes of dementia could lead to clues about the primary pathogenesis of different forms of dementia, which remains unknown.

Specific LDs are common (i.e., 5–15% of children) and present early in life with difficulties with word decoding / fluency, reading comprehension, spelling, writing, number sense or mathematical reasoning, despite overall normal or high levels of intelligence [13]. Specific LDs fall under the umbrella category of Neurodevelopmental Disorders in the new Diagnostic and Statistical Manual of Mental Disorders Version 5 (DSM-5). Specific LDs should not be confused with intellectual disability / intellectual development disorder, which is a separate diagnostic entity within Neurodevelopmental Disorders. Intellectual Disability has onset during the childhood or adolescent years and includes both intellectual and adaptive functioning deficits in conceptual, social and practical domains; these conditions are sometimes co-morbid with LD [14]. By contrast, specific LD refers to handicaps that are not related to physical, developmental or intellectual disabilities.

Three studies have suggested an association between LDs and specific dementia types [1–3]. The first study revealed that a PPA cohort reported increased family history of developmental dyslexia but this cohort was not broken down into subtypes of PPA. The second study found that the association was only present within the L-PPA subtype, which is L-PPA frequently associated with underlying AD pathology [15, 16]. However, the autopsy follow-up of the patients from the first study demonstrated that both AD and FTLD pathology was present in the PPA cohort reporting family history of childhood LD [3]. It remains unknown whether adults with childhood LD are in fact at increased risk of atypical phenotypes of dementia, and if so, which dementias.

The objective of this clinic-based, pilot study was to further our understanding of the association between self-reported LDs and three forms of atypical dementia that are often (though not always) associated with underlying AD pathology (L-PPA, PCA, Dysexecutive AD). We hypothesized that a higher proportion of individuals diagnosed with atypical dementias, vs. Typical AD, would report a positive childhood history of LD. Both LDs and atypical dementias are associated with atypical, long-range brain network topography [9, 17–20], providing a potential common mechanism for LD and atypical dementia pathogenesis. Further evidence in support of the hypothesis that developmental trajectories influence neurodegenerative phenotypes includes several studies demonstrating childhood and adolescent brain differences in people at genetic risk for Typical AD [21–26].

We built on the prior studies by grouping atypical dementia patients together who had a high likelihood of underlying AD pathology, by considering personal, rather than family history, of childhood LD, and by adjusting for several potential confounders.

Ultimately, this line of research could identify a new risk factor for atypical dementia, which might lead to clues about the cause of dementia or AD (which remains unknown) and to the development of preventative interventions for atypical dementias, which remain unavailable.

## Materials & Methods

The Columbia University Medical Center Internal Review Board approved this study. This study was a retrospective chart review. All patient records/information was anonymized and de-identified prior to analysis. As such, study participants were not required to provide informed consent for their clinical records to be used in this study.

### Initial Case Ascertainment

The authors reviewed consecutive neuropsychological reports of patients evaluated by three neuropsychologists at the Columbia University Memory Disorders Clinic from March 10^th^, 2011 through May 20^th^, 2014. Patients were referred from the Columbia University faculty practice and New York State Psychiatric Institute Memory Disorders clinic. Consecutive reports were reviewed in order to reduce selection bias.

Patients who were age 40 through 80 years at initial or follow-up evaluation by the neuropsychologist and suspected clinically by the neuropsychologist after complete neuropsychological examination to have any diagnosis of Early Onset AD, Typical AD, Atypical Dementia of any type (including PCA), PPA of any type, or behavioral variant FTD (bvFTD) were initially selected. Age 80 was chosen as the cutoff to minimize recall bias and to maintain comparability with the Atypical Dementia and FTLD patients. Patients who were born and raised outside the United States were excluded because patients from countries in which diagnostic rates for children with LDs lag behind the U.S. would be more likely to report negative history of childhood LD and also because language barriers cause diagnostic challenge in atypical dementias. Patients diagnosed with Dementia with Lewy Bodies, Parkinson’s Disease Dementia, another movement disorder (Progressive Supranuclear Palsy, Corticobasilar Syndrome / Degeneration, “Parkinson’s Plus”, motor neuron disease, normal pressure hydrocephalus) or with documented evidence of symptoms of a movement disorder were excluded because is actually a heterogeneous group of dementias that are difficult to diagnose accurately. We made further exclusions if there was documented evidence of prior traumatic brain injury, major medical illness (e.g. cancer, heart surgery, traumatic brain injury, brain tumor, multiple sclerosis), and stroke (as documented in past medical history by evaluating neuropsychologist).

**Fig 1:**
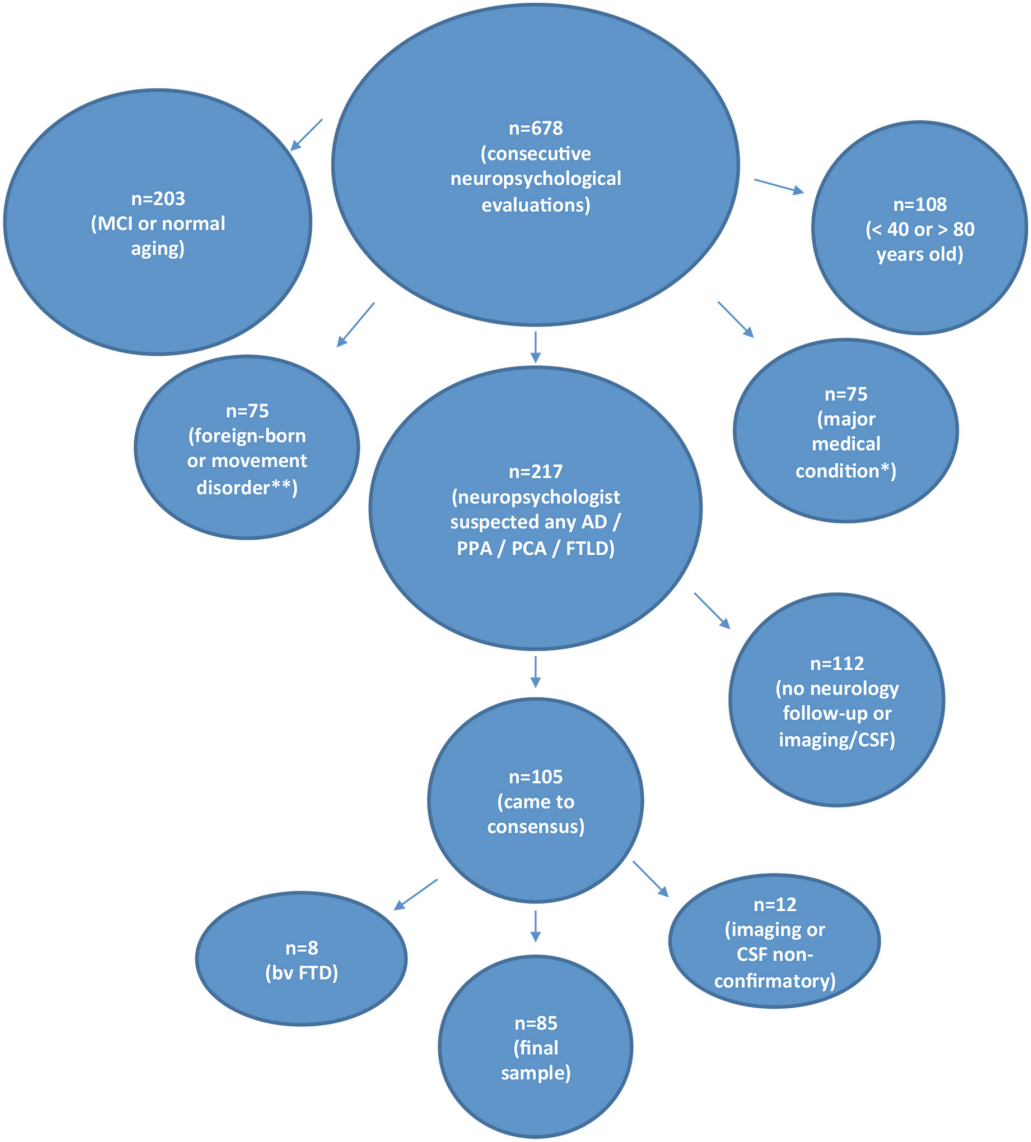
Study Flow Chart with Inclusion and Exclusion Criteria. *e.g., cancer, heart surgery, traumatic brain injury, brain tumor, multiple sclerosis, stroke (as documented by evaluating neuropsychologist). **e.g., myoclonus, dystonia, parkinsonism.

Fig 1 summarizes how the final study sample was obtained. Of the initial 678 consecutive neuropsychological evaluations, 217 were suspected by the neuropsychologist to have any type of neurodegenerative dementia, including AD, PPA, PCA or FTLD-related syndromes. Of these, 112 cases were further excluded if both radiology and CSF data were unavailable or if there was no neurology follow-up visit.

Subsequently, 105 cases diagnosed by the treating neuropsychologist with Late-onset Typical AD, Early-onset AD, Atypical AD (including PCA and Dysexecutive AD), PPA and FTLD-related syndromes were subjected to a consensus diagnostic process. For late-onset AD patients, consensus was considered present if the diagnosing neuropsychologist and the treating neurologist both documented a diagnosis of Typical, late-onset AD. For all other cases, including early onset AD cases, two neuropsychologists and one neurologist specializing in Aging & Dementia conferenced the patients one by one, using the appropriate published criteria, as described below. During this process, the case reviewers were blinded to the Social & Developmental History within the neuropsychological report, which contained information about LDs. After consensus, 12 patients were further excluded because neuroimaging or CSF was inconsistent with AD and also inconsistent with FTLD. 8 patients with bvFTD were excluded to allow for a simple comparison between two groups (Atypical Dementia and Typical AD). 2 cases with non-fluent PPA were excluded because neuroimaging was inconclusive (global hypoperfusion) and CSF was unavailable. The 85 remaining patients were grouped into the comparison groups shown in Table 1.

**Table 1:** Patient Characteristics at Baseline Neuropsychological Evaluation.

Characteristic	Typical AD (n = 68)	Atypical AD (n = 17)	p-value
Age at evaluation, y, mean (SD)	70.1 (6.7)	67.1 (8.0)	0.17
Right Handedness, n (%)	62 (91%)	13 (77%)	0.09
Female, n (%)	37 (54%)	7 (41%)	0.32
Education, y, mean (SD)			
College Education or higher, n (%)	43 (63%)	13 (77%)	0.30
Family History^a^, n (%)	29 (43%)	6 (35%)	0.58
Probable LD, n (%)	6 (9%)	5 (29%)	0.02
Symptom duration^b^, y, mean (SD)	3.3 (2.3)	4.1 (2.0)	0.05
Supportive Data, n (%)			
Neuroimaging	68 (100%)	17 (100%)	—
CSF	8 (12%)	2 (12%)	—

^a^First degree family member with any type of late-life cognitive impairment

^b^Years from first symptom onset to age at evaluation

### Comparison Groups

The “Atypical Dementia” group included anyone with a consensus diagnosis of L-PPA, PCA, or Dysexecutive AD. The Typical AD group included patients with consensus diagnosis of Typical AD (with onset at any age).

#### Atypical Dementia

L-PPA was diagnosed using the clinical criteria by Gorno-Tempini, et al [27]. These criteria allow for clinical, radiologically-confirmed, and pathologically-confirmed levels of classification. Patients without radiological or CSF supportive data were excluded. Of note, up to 31% of patients with PPA of any type may not classify neatly into one or another category [28]. In this study, we observed one instance in which a PPA patient simultaneously fulfilled clinical criteria for more than one type of PPA, but this patient was not included in the final analysis due to lack of imaging or CSF data.

PCA was diagnosed if the patient presented with prominent, progressive visuospatial, visuoperceptual, literacy, or praxic skills, with radiological evidence of parietal, occipital, or occipitotemporal cortices, relative sparing of the medial temporal cortex, and/or evidence of amyloid/tau in the CSF. Diagnostic criteria for PCA are currently under development [29].

Dysexecutive AD was distinguished from Typical AD using a method previously described which relies on neuropsychological test data [8, 30]. Specifically, patients who were initially diagnosed by the neuropsychologist with AD with a dysexecutive or frontal presentation were given a memory score (delayed recall subtracted from immediate recall in the logical memory test) and an executive score (Trail Making Test B minus Trail Making Test A). Z-scores were calculated for executive and memory function. Participants with executive function scores at least one standard deviation below memory scores were confirmed as belonging to the Dysexecutive AD subgroup. If the patient was severely impaired in both, clinical history was used to confirm that dysexecutive symptoms were most prominent at onset.

Reference ranges for CSF confirmation of AD pathology were as follows: 0–210 ng/l for tau protein, 400–1200 pg/l for beta amyloid, 0–0.5 for tau/beta index. Imaging findings were confirmed using the written report by the radiologist and the documented interpretation by treating neurologist. Asymmetric temporoparietal atrophy or hypometabolism on structural or functional neuroimaging, respectively, was considered as support for L-PPA. Predominant frontotemporal atrophy, without significant parietal, temporal or occipital hypometabolism, was considered as not consistent with L-PPA type, per the accepted criteria.

#### Typical AD

Typical AD was diagnosed using the NINCDS-ADRDA Criteria [31]. Presence of typicality of symptoms (primarily amnestic) was confirmed by methods previously described to confirm the Dysexecutive subgroup. Supportive evidence of underlying AD pathology was required for all cases (predominant temporo-parietal hypometabolism or atrophy) as documented either by the radiology report or the treating clinicians. Cases of early-onset AD (defined as symptom onset prior to age 60) were included in the Typical AD group because it was important to determine whether these cases belonged to Typical AD or Atypical AD groups based on focality of presentation, irrespective of age.

#### Frontotemporal Lobar Degeneration Syndromes

Cases of bvFTD, aPPA, and SD were initially identified and included in a group labeled “FTLD”. Although these can have differing underlying pathologies, they all have in common non-AD related pathology in the vast majority of cases [32]. Behavioral variant FTLD was diagnosed using criteria by Rascovsky et al [33]. Non-fluent/agrammatic PPA and Semantic Dementia were diagnosed using the criteria by Gorno-Tempini, et al [27]. Due to low sample size, the FTLD group was not included in the final analysis. The primary analysis was between Atypical Dementia group and Typical AD group.

### Childhood LD

The standard assessment by neuropsychologists for patients with dementia routinely includes inquiry about history of childhood LDs. For this study, history of childhood LD was affirmed using a consensus review of the Social & Developmental History (between two neuropsychologists). Self-reported LD was categorized as: Probable, Possible or Absent. Probable LD was assigned if the patient reported previously being formally diagnosed or if there were two or more documented statements in the Educational History supporting presence of LD. These included: childhood need for special class, need for therapy, significant inattention or hyperactivity, difficulty with reading, spelling, or letter direction, poor overall school performance, and significant difficulty with math relative to reading. Possible LD was diagnosed if only one documented statement appeared in the history. Although no validated scale was used in this study, many of these items are included in the Colorado Learning Difficulties Questionnaire, which has been previously validated [34]. We categorized subjects as having language-related disabilities (e.,g., reading, writing, speaking) or non-language-related disabilities (e.g., math, numbers) but no specific diagnoses of LDs were made.

### Co-Variates

Age of initial diagnosis and age of onset of first symptoms were obtained from the clinical evaluation at which the patient was first diagnosed. Level of education was ascertained by self-report and treated categorically in the final analysis (continuously in sensitivity analyses). First degree family history of any type of cognitive impairment was also ascertained by self-report. Cognitive impairment of any type was used as a surrogate for positive family history of dementia. Ethnicity information was not recorded because it was not readily or accurately available per the notes for many of the patients.

### Data Analysis

85 patients were included in the final analysis (Typical AD n = 68, Atypical Dementia n = 17).

All comparisons, including the primary analysis, were made between the Typical AD and Atypical Dementia group. Chi-squared tests were used to compare categorical variables (handedness, gender, college degree, first degree family history) and t-tests were used to compare continuous variables (age, symptom duration).

In the primary analysis, we performed a logistic regression with Atypical Dementia vs. Typical AD, as the outcome, and Probable LD vs. Absent or Possible LD, as the primary predictor (Model 1). Model 2 was further adjusted for age, gender, education (college degree or higher), and handedness. To account for disease stage, Model 3 was further adjusted for symptom duration. Symptom duration was used as a proxy for disease stage because the use of cognitive test scores or CDR scores would have created difficultly considering that minor language or vision disturbances in Atypical Dementia can cause disproportionately lower scores on cognitive or functional tests.

We also investigated whether first degree family history of cognitive impairment or dementia of any kind influenced this association by performing separate analyses in patients with and without family history. Finally, we explored qualitatively whether the type of LD (language vs. non-language) segregated with the type of Atypical Dementia and explored whether early-onset AD cases differed in frequency of self-reported LD from the other group. To ensure validity of findings given sample size, we calculated Nagelkerke and Cox & Snell coefficients and Chi-square values of significance for each model. We also conducted bootstrap analyses to address small sample size and to confirm the validity of the findings. Our bootstrapping sampling method was at 90% resampling at each iteration. Data were analyzed using IBM SPSS version 21.

## Results

85 patients were included in the final analysis (Typical AD, n = 68, Atypical Dementia, n = 17). The 17 cases of Atypical Dementia included 11 L-PPA patients, 3 PCA patients and 3 Dysexecutive AD patients. Overall, there were no significant differences in age at evaluation, symptom duration, handedness, sex, and educational levels between the Atypical Dementia group and Typical AD group, although there was a trend towards higher percentage of left-handedness and longer symptom duration prior to diagnosis in the Atypical Dementia group. Structural or functional imaging was available for 100% of Typical AD and 100% of Atypical Dementia cases; CSF was available for 12% of Typical AD and 12% of Atypical Dementia cases. In both cases of L-PPA who underwent CSF testing, CSF was positive for AD biomarkers. All 11 cases of L-PPA had radiology testing consistent with Probable L-PPA subtype.

In logistic regression models fully adjusted for age at evaluation, sex, handedness, education and symptom duration (see Table 2), patients with Probable LD, compared to a combined group of patients with Possible or Absent LD, were significantly more likely to be diagnosed with Atypical Dementia vs. Typical AD (OR 13.1, 95% CI 1.3–128.4). Four subjects reported Possible LD and were included in the Possible/Absent group. In qualitative observations regarding type of LD, we noted that of the three L-PPA cases with Probable LD, all three involved language difficulty. By contrast, both PCA cases reporting Probable childhood LD endorsed difficulty with attention and math (as did another PCA case with Possible LD, see Table 3). There were no apparent interactions with self-reported family history of dementia.

**Table 2:** Logistic regression analyses, Self-reported LD and Atypical AD (vs. Typical AD).

Self-Reported Childhood Learning Disability	Model 1^a^ OR (95% CI)	Model 2^b^ OR (95% CI)	Model 3^c^ OR (95% CI)
**Absent or Possible LD**	—	—	—
**Probable LD**	4.3 (1.1–16.4)	13.7 (1.4–134.5)	13.1 (1.3–128.4)

^a^Unadjusted

^b^Adjusted for Age at Evaluation, Gender, Handedness and Education

^c^Additionally adjusted for symptom duration

**Table 3:** Characterization of subjects with Possible or Probable LD.

Gender	Hand Preference	LD Type	Age of onset	First symptom	Consensus Diagnosis^a^
***Probable LD:***					
Female	Right	Dyslexia / ADHD	55	Mixed	Typical AD
Female	Right	Dyslexia / ADHD	66	Memory	Typical AD
Male	Right	Dyslexia	70	Judgment/problem solving	Typical AD
Male	Right	Dyslexia	67	Attention	Typical AD
Male	Right	ADHD	61	Memory	Typical AD
Male	Right	Dyslexia	53	Mixed	Typical AD
Female	Left	Dyslexia	48	Language	L-PPA
Male	Left	Dyslexia	67	Language	L-PPA
Male	Right	Stuttering	61	Language	L-PPA
Female	Right	ADHD	67	Mixed	PCA
Male	Right	Dyscalculia	62	Visuospatial	PCA
***Possible LD:***					
Female	Right	Dyslexia	73	Memory	Typical AD
Male	Right	Stuttering	72	Memory	Typical AD
Male	Right	ADHD	49	Behavioral	Dysexecutive AD
Female	Right	Dyscalculia	64	Attention	PCA

^a^L-PPA = logopenic-type Primary Progressive Aphasia; PCA = Posterior Cortical Atrophy

In sensitivity analyses, when we considered Possible and Probable LD together (vs. Probable LD only), and when we treated education as a categorical or continuous variable, results were unchanged. When we included subjective report of first degree family history of any cognitive impairment in the model as a co-variate rather than as an interaction term, results did not change. Statistical tests performed due to small sample sizes confirmed the validity of these findings. For each model, correlation coefficients for exact tests were small, indicating low degree of inter-variable correlation. For each model, the bootstrapped confidence intervals were more narrow and retained significance (CI 0.2 to 22.3). To identify why the OR changed from Model 1 to Model 2, we used step-wise analyses which identified that addition of the education variable (College or Higher vs. Less than College) to the model was responsible for this change.

## Discussion

In this clinic-based study of 85 patients with consensus diagnoses of Atypical Dementia or Typical AD supported by radiological or CSF data, patients who presented for neuropsychological evaluation who also reported positive childhood history of LD were more likely to be diagnosed with Atypical Dementia vs. Typical AD, compared with subjects reporting no childhood history of LD. Results remained significant after adjusting for age, sex, handedness, education and symptom duration. Qualitative observation revealed that the three subjects reporting a history of LD in the L-PPA group endorsed difficulty with language while both subjects reporting an LD history in the PCA group described difficulty with attention and/or numbers, supporting the notion that childhood cognitive phenotypes may predict phenotypic patterns of neurodegenerative disease.

The present study confirms and contributes to the three prior studies on this subject. In a study of over 600 patients from the Northwestern Alzheimer’s Disease Center registry, 16% of 108 individuals with PPA (of any type) and 32% of their first degree family members answered affirmatively to having a history of LD [1]. These frequencies were significantly higher than those noted in control patients without AD, patients with typical amnestic AD, and patients with behavioral variant FTLD. Among the families of PPA probands, clusters of LDs, particularly developmental dyslexia, were noted. A second study utlized a more specific breakdown of the type of PPA, with imaging-supported classification [2]. Specifically, 8% of all PPA patients from the University of California San Francisco Memory and Aging Center had self- or informant-based report personal history of delay in speaking or reading at baseline medical interview. This finding was driven by a particularly high percentage (25%) in the 48 patients with L-PPA. Radiologic data in, L-PPA patients with LD was notable for atrophy in the same areas affected as those in developmental dyslexia (posterior middle and superior temporal gyri). The clinical specificity of LD to L-PPA may indicate an association with AD pathology. Another group which studied a cohort of individuals with PPA of any type found both AD and FTLD pathology, suggesting that any specific association with LD may only have relevance in predicting clinical phenotype but not necessarily underlying pathology [3]. In our study, 0/8 behavioral variant FTLD patients reported Probable LD. Due to low sample size, there were no aPPA or SD cases which made the final analysis, so we were unable to assess whether the association between LD and PPA is specific to L-PPA type, which remains to be further studied [2]. Our study is the first to suggest that Posterior Cortical Atrophy may be related to developmental non-language LD’s (i.e., dyscalculia).

**Fig 2:**
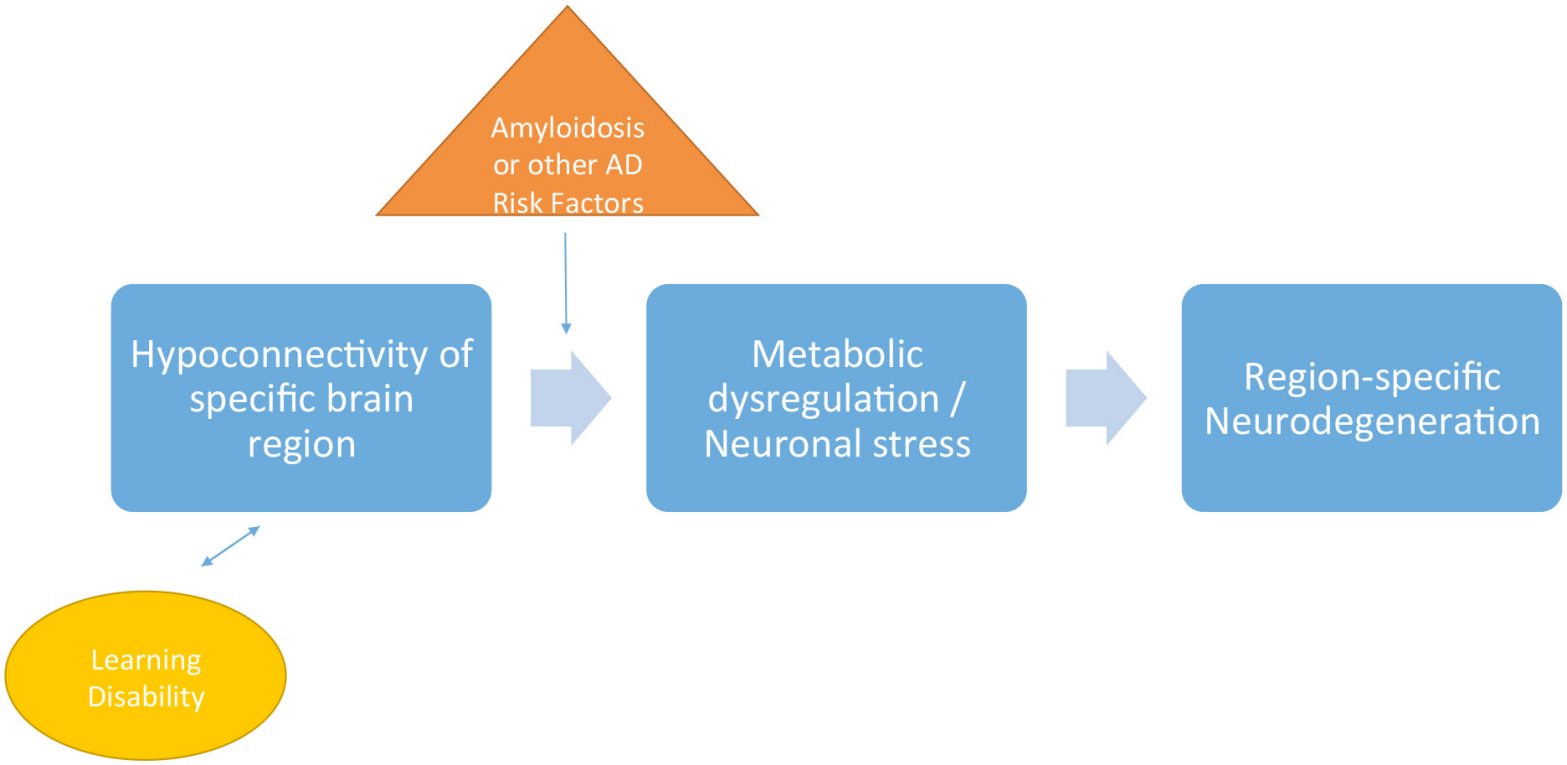
Conceptual Map of Pathological Links Between Developmental Learning Disabilities and Region-Specific Neurodegeneration. In the model shown above, atypical connectivity associated with learning disabilities, in the presence of aging and other dementia risk factors, serves as the primary impetus for metabolic failure in the related brain network, which subsequently leads to region-specific neurodegenerative pathology.

Impaired connectivity within a specific brain region, from genetic or acquired causes, could represent the shared pathological mechanism linking LDs to Atypical AD or other dementias. This hypothesis is sometimes referred to as “selective vulnerability”. Fig 2 presents a simplified model of this conceptual framework. Region-specific connectivity differences associated with LDs [20] could predispose to neuronal failure in the face of amyloidosis or other dementia risk factors, thus commencing the neurodegenerative pathophysiological cascade. Connectivity can be impaired at the synaptic, dendritic, axonal or neuronal level. In fact, recent evidence may support the notion that disruptions at the synaptic level by soluble amyloid represent primary events in AD neurodegeneration [35, 36]. Trophic disconnections may be central to neurodegeneration due to AD [20][37, 38].

The conflicting findings regarding whether developmental dyslexia is associated specifically with L-PPA, and not other subtypes of PPA, are especially important because logopenic-type PPA is frequently associated with underlying AD pathology [15, 16]. This implies that the relationship between LDs and dementia could be specific to AD. AD, unlike other dementias, is thought to begin specifically within associational cortex of the brain; tau-related neurodegeneration in AD *selectively* affects long, projection neurons which connect higher association cortex with other brain regions [3]. Developmental LDs are also characterized pathologically by impairments in long-range brain network connections and differences in left-right white matter dominance. If some cases of Atypical AD represent downstream extensions of LD, then Typical AD might also represent a direct extension of a neurodevelopmental phenotype. In fact, the literature on Typical AD and neurodevelopment supports this. In Typical AD, the lateral entorhinal cortex, precuneus and posterior cingulate gyrus appear the most vulnerable to neurodegeneration. During typical neurodevelopment, these areas are the last to myelinate and the most poorly myelinated [4]. Differences in white matter connectivity, mitochondrial activity and gray matter volume in these regions have been demonstrated in infants, children, adolescents and non-demented persons at genetic risk for Typical AD (due to *APOE4* or *SORL1* variants) [21–26]. Also, proteins involved in AD-related neurodegeneration function in a variety of neurodevelopmental processes [39, 40]. With this in mind, genes found to be associated with LDs should continue to be investigated as candidate genes for Atypical AD or other dementias [41, 42].

Small sample size, selection bias, poor measurement of the primary risk factor (LD), and suboptimal accounting of disease severity, among other limitations to this study, could have negatively influenced the results. The authors carefully discussed these potential limitations prior to selection of the final study design. Only 85 of 678 patients (14%) were included in the final analysis. Small sample size could have introduced error into the analysis. Nonetheless, the large effect sizes observed, the retained significance in bootstrapped analyses, as well as the retained significance across other sensitivity analyses, would suggest that the finding is in fact not spurious. In addition, using exact tests, we demonstrated that there was not high inter-correlation of the variables, which also supports the validity of the study in the context of small sample size. Sensitivity analyses also revealed that the small sample size and unequal distribution of education could have explained the change in OR from Model 1 to Model 2 (we did not have years of education, in a continuous variable, available for analysis.) Regarding selection bias, exclusion of patients with non-neurodegenerative dementias, such as those with migraine or psychiatric illness, was not expected to cause bias in one direction or another. In fact, consecutive review of all cases was done specifically to mimimize selection bias. Another major limitation of this study involved the collection of the data on childhood history of LD. With only retrospective data, we relied on self-report of LDs and the style of elicitation of information about LDs varied by neuropsychologist. We could not objectively confirm presence or absence of LD using neuropsychological data because patients were already demented. Additionally, patients with a diagnosis of Atypical AD may have been more likely to answer affirmatively to questions about history of LDs because they were seeking an explanation for their unique symptoms. According to this logic, patients with early onset AD and FTLD would also be expected to answer affirmatively but we did not note increased frequency of LDs in these groups (1/10 patients with early-onset AD reported probable LD). Other limitations included reliance on symptom duration as a proxy for disease severity and being unable to account for severity of LD. Severity of underlying AD or LD pathology could influence the relationship between LD and AD phenotype. Additionally, we could not accurately collect information on ethnicity. Ethnicity is an important potential confounder because diagnostic rates of LDs and of AD vary by ethnicity. A related limitation was that our final sample was demographically homogenous, limiting the generalizability of our findings. Lastly, while we were able to draw conclusions about Atypical Dementia as a whole, this study was underpowered to evaluate subtypes of Atypical Dementia and to draw direct conclusions about the association between LDs, AD and FTLD.

Despite the limitations, the paper has several strengths. One important strength of this study was the availability of structural or functional imaging for all cases where available to ensure, to the best of our abilities, little to no pathological overlap between groups. We limited the study to patients with supportive data because significant discordance exists between clinical phenotypes of dementia and underlying cellular pathology. The clinical classifications for PPA, for example, require further refinement, as patients can sometimes fulfill more than one or neither clinical diagnostic classification for PPA [43]. We grouped the patients such that patients included in the Atypical Dementia group were likely (to the extent possible) to have underlying AD pathology as a common factor. We attempted, in essence, to create a group of Atypical AD patients. By doing this, we found a 30% frequency of self-reported LD, which is consistent with the 25% frequency of self-reported learning disabilities in patients with L-PPA. None of the patients in our L-PPA group had frontotemporal hypometabolism on functional imaging, and all had temporoparietal metabolism, which is much more strongly associated with AD pathology. Another important strength includes the fact that we adjusted for several potential confounders.

## Conclusions

During the initial neuropsychological evaluation, patients diagnosed with Atypical Dementias (i.e., L-PPA, PCA, or Dysexecutive AD) endorse symptoms of previous childhood LD at a higher rate than patients diagnosed with Typical AD. Specifically, some patients with PPA endorse childhood symptoms of language difficulty and some patients with PCA endorse childhood symptoms of math and/or attention difficulty. In the near term, studies are required to confirm these findings using more accurate measurement of childhood LD and pathologically-confirmed diagnoses of neurodegenerative disease. If the association between LD and Atypical Dementia is confirmed, future longitudinal studies will be required to identify whether there is underlying common pathology between LD and dementia related to network connectivity. Future research is also needed in order to determine the probable genetic and/or modifying factors (and the underlying mechanisms) that predispose some, but not all people, with atypical neurodevelopment, to develop atypical neurodegenerative disease.
